# Quinoa Seed Quality Response to Sodium Chloride and Sodium Sulfate Salinity

**DOI:** 10.3389/fpls.2016.00790

**Published:** 2016-06-03

**Authors:** Geyang Wu, Adam J. Peterson, Craig F. Morris, Kevin M. Murphy

**Affiliations:** ^1^School of Food Science, Washington State UniversityPullman, WA, USA; ^2^Department of Crop and Soil Sciences, Washington State UniversityPullman, WA, USA; ^3^United States Department of Agriculture/Agricultural Research Service-Western Wheat Quality Laboratory, Washington State UniversityPullman, WA, USA

**Keywords:** quinoa, soil salinity, protein content, hardness, density

## Abstract

Quinoa (*Chenopodium quinoa* Willd.) is an Andean crop with an edible seed that both contains high protein content and provides high quality protein with a balanced amino acid profile in embryonic tissues. Quinoa is a halophyte adapted to harsh environments with highly saline soil. In this study, four quinoa varieties were grown under six salinity treatments and two levels of fertilization, and then evaluated for quinoa seed quality characteristics, including protein content, seed hardness, and seed density. Concentrations of 8, 16, and 32 dS m^-1^ of NaCl and Na_2_SO_4_, were applied to the soil medium across low (1 g N, 0.29 g P, 0.29 g K per pot) and high (3 g N, 0.85 g P, 0.86 g K per pot) fertilizer treatments. Seed protein content differed across soil salinity treatments, varieties, and fertilization levels. Protein content of quinoa grown under salinized soil ranged from 13.0 to 16.7%, comparable to that from non-saline conditions. NaCl and Na_2_SO_4_ exhibited different impacts on protein content. Whereas the different concentrations of NaCl did not show differential effects on protein content, the seed from 32 dS m^-1^ Na_2_SO_4_ contained the highest protein content. Seed hardness differed among varieties, and was moderately influenced by salinity level (*P* = 0.09). Seed density was affected significantly by variety and Na_2_SO_4_ concentration, but was unaffected by NaCl concentration. The samples from 8 dS m^-1^ Na_2_SO_4_ soil had lower density (0.66 g/cm^3^) than those from 16 dS m^-1^ and 32 dS m^-1^ Na_2_SO_4_, 0.74 and 0.72g/cm^3^, respectively. This paper identifies changes in critical seed quality traits of quinoa as influenced by soil salinity and fertility, and offers insights into variety response and choice across different abiotic stresses in the field environment.

## Introduction

Quinoa (*Chenopodium quinoa* Willd.) has garnered much attention in recent years because it is an excellent source of plant-based protein and is highly tolerance of soil salinity. Because soil salinity affects between 20 and 50% of irrigated arable land worldwide (Pitman and Läuchli, 2002), the question of how salinity affects seed quality in a halophytic crop like quinoa needs to be addressed. Protein content in most quinoa accessions has been reported to range from 12 to 17%, depending on variety, environment, and inputs (Rojas et al., 2015). This range tends to be higher than the protein content of wheat, barley, and rice, which were reported to be 10.5–14%, 8–14%, and 6–7%, respectively (Orth and Shellenberger, 1988; Shih, 2006; Cai et al., 2013). Additionally, quinoa has a well-balanced complement of essential amino acids. Specifically, quinoa is rich in lysine, which is considered the first limiting essential amino acid in cereals (Taylor and Parker, 2002). Protein quality such as Protein Efficiency Ratio is similar to that of casein (Ranhotra et al., 1993). Furthermore, with a lack of gluten protein, quinoa can be safely consumed by gluten sensitive/intolerant population (Zevallos et al., 2014).

Quinoa shows exceptional adaptation to harsh environments such as drought and salinity (González et al., 2015). Soil salinity reduces crop yields and is a worldwide problem. In the United States, approximately 2.2 million hectares of cropland in 48 States were occupied by saline soils, while another 30.8 million hectares are at risk of becoming saline (United States Department of Agriculture [USDA], 2011). The salinity issue leads producers to grow more salt-tolerant crops, such as quinoa.

Many studies have focused on quinoa’s tolerance to soil salinity, with a particular emphasis on plant physiology (Ruiz-Carrasco et al., 2011; Adolf et al., 2012; Cocozza et al., 2013; Shabala et al., 2013) and agronomic characteristics such as germination rate, plant height, and yield (Prado et al., 2000; Chilo et al., 2009; Razzaghi et al., 2012; Peterson, 2013; Peterson and Murphy, 2015). For instance, Razzaghi et al. (2012) showed that the seed number per m^2^ and seed yield did not decrease as salinity increased from 20 to 40 dS m^-1^ in the variety Titicaca. Ruiz-Carrasco et al. (2011) reported that under 300 mM NaCl, germination and shoot length were significantly reduced, whereas root length was inhibited in variety BO78; variety PRJ biomass was less affected and exhibited the greatest increase in proline concentration. Jacobsen et al. (2000) suggested that stomatal conductance, leaf area, and plant height were the characters in quinoa most sensitive to salinity. Wilson et al. (2002) examined salinity stress of salt mixtures of MgSO_4_, Na_2_SO_4_, NaCl, and CaCl_2_ (3 – 19 dS m^-1^). No significant reduction in plant height and fresh weight were observed. In a comparison of the effects of NaCl and Na_2_SO_4_ on seed yield, quinoa exhibited greater tolerance to Na_2_SO_4_ than to NaCl (Peterson and Murphy, 2015).

Few studies have focused on the influence of salinity on seed quality in quinoa. Karyotis et al. (2003) conducted a field experiment in Greece (80 m above sea level, latitude: 39.7°N). With the exception of Chilean variety ‘No. 407,’ seven other varieties exhibited significant increases in protein (13–33%) under saline-sodic soil, with electrical conductivity (EC) of 6.5 dS m^-1^. Seed minerals contents of phosphorous, iron, copper, and boron did not decrease under saline conditions. Koyro and Eisa (2008) found a significant increase in protein and a decrease in total carbohydrates under high salinity (500 mM). Increasing total soluble sugar, sucrose, and glucose were observed under salinity stress because of starch hydrolysis (Ruffino et al., 2010). The same study found a significant decrease in lipids and relative water content under salinity. Pulvento et al. (2012) indicated that fiber and saponin contents increased under saline conditions with well water/sea water ratio of 1:1 compared to those under non-saline soil.

Protein is one of the most important nutritional components of quinoa seed. The content and quality of protein contribute to the nutritional value of quinoa. Additionally, seed hardness is an important trait in crops such as wheat and soybeans. For instance, kernel hardness highly influences wheat end-use quality (Morris, 2002) and correlates with other seed quality parameters such as ash content, semolina yield, and flour protein content (Hrušková and Švec, 2009). Hardness of soybean influenced water absorption, seed coat permeability, cookability, and overall texture (Zhang et al., 2008). Quinoa seed hardness was correlated with the texture of cooked quinoa, influencing hardness, chewiness, and gumminess, and potentially consumer experience (Wu et al., 2014). Furthermore, seed density is also a quality index and is negatively correlated with the texture of cooked quinoa such as hardness, cohesiveness, chewiness, and gumminess (Wu et al., 2014). Hence, protein content, seed hardness, and seed density were selected as indexes of quinoa seed quality in this study.

Chilean lowland varieties have been shown to be the most well-adapted to temperate latitudes (Bertero, 2003), and therefore they have been extensively utilized in quinoa breeding programs in both Colorado State University and Washington State University (Peterson and Murphy, 2015). The previous study found the varieties of CO407D, UDEC-1, Baer, and QQ065 exhibited extremely high tolerance to Na_2_SO_4_ and relatively high tolerance to NaCl in terms of agronomic performance such as yield, plant height, and leaf greenness (Peterson and Murphy, 2015). However, quinoa seed quality under salinity stress remains to be evaluated, since seed quality is critical to nutrition value as well as consumers’ liking of the product. Hence, the objectives of this study were to (1) examine the effect of soil salinity on the protein content, seed hardness, and density of quinoa varieties, (2) determine the effect of different levels of two agronomically important soil salts, NaCl and Na_2_SO_4_, on seed quality, (3) determine the different influence of and NaCl and Na_2_SO_4_; and (4) test the influence of fertilization level on salinity tolerance of quinoa.

## Materials and Methods

### Genetic Material

Quinoa germplasm was obtained from Dr. David Brenner at the USDA-ARS North Central Regional Plant Introduction Station in Ames, IA, USA. The four quinoa varieties, CO407D (PI 596293), UDEC-1 (PI 634923), Baer (PI 634918), and QQ065 (PI 614880), were originally sourced from lowland Chile. CO407D was released by Colorado State University in 1987. UDEC-1, Baer, and QQ065 were varieties from northern, central, and southern locations in Chile with latitudes of 34.63° S, 38.70° S, and 42.50° S, respectively.

### Experimental Design

A controlled environment greenhouse study was conducted using a split-split-plot randomized complete block design with three replicates per treatment. Factors included four quinoa varieties, two fertility levels, and seven salinity treatments (three concentration levels each of NaCl and Na_2_SO_4_). Three subsamples, each representing a single plant, were evaluated for each treatment combination. Quinoa variety was treated as the main plot, salinity level as the sub-plot, and fertilization as the sub-sub-plot. Salinity levels included 8, 16, and 32 dS m^-1^ of NaCl and Na_2_SO_4_. The details of controlling salinity levels were described by Peterson and Murphy (2015). In brief, fertilization was provided by a mixture of alfalfa meal, monoammonium phosphate, and feather meal. The low fertilization level included 1 g of N, 0.29 g of P, and 0.29 g of K per pot; and the high fertilization level included 3 g of N, 0.86 g of P, and 0.86 g of K per pot. Each pot contained about 1 L of Sunshine Mix #1 (Sun Gro Horticulture, Bellevue, WA, USA) (dry density of 100 g/L, water holding capacity of ca. 480 g/L potting mix). The entire experiment was conducted twice, with the planting dates of September 10th, 2011 and October 7th, 2011.

### Seed Quality Tests

Quinoa was harvested at maturity. As described in the previous study (Peterson and Murphy, 2015), seeds were first stripped by hand from the inflorescences, and then threshed in a single-head thresher (Precision Machine Company, Lincolin, NE, USA). The resulting material was cleaned using a Clipper Office Tester (Seedburo, Des Plaines, IL, USA) before the seed quality tests.

Protein content of quinoa was determined using the Dumas combustion nitrogen method (LECO Corp., Joseph, Mich., USA) (AACCI Method 46-30.01) (AACC International, 1995). A factor of 6.25 was used to convert nitrogen to protein. Seed hardness was determined using the Texture Analyzer (TA-XT2i) (Texture Technologies Corp., Scarsdale, NY, USA) and a modified rice kernel hardness method (Krishnamurthy and Giroux, 2001). A single quinoa kernel was compressed until the point of fracture using a 1 cm^2^ cylinder probe traveling at 5 mm/s. Repeat measurements were taken on nine random kernels. The seed hardness was recorded as the average peak force (kg) of the repeated measures.

Seed density was determined using a pycnometer (Pentapyc 5200e, Quantachrome Instruments, Boynton Beach, FL, USA). Quinoa seed was placed in a closed micro container, and compressed nitrogen was suffused into the container. Pressure in the container was recorded both with and without nitrogen. The volume of the quinoa sample was calculated by comparing the standard pressure obtained with a stainless steel ball. Density was the seed weight divided by the displaced volume. Seed density was collected on only the second greenhouse experiment.

### Statistical Analysis

Data were analyzed using the PROC GLM procedure in SAS (SAS Institute, Cary, NC, USA). Greenhouse experiment repetition was treated as a random factor in protein content and seed hardness analysis. Variety, salinity, and fertilization were treated as fixed factors. Fisher’s LSD Test was used to access multiple comparisons. Pearson correlation coefficients between protein, hardness, and density were obtained via PROC CORR procedure in SAS, using the treatment means.

## Results

### Protein

Variety, salinity, and fertilization all exhibited highly significant effects on protein content (*P* < 0.001) (**Table 1**). The greatest contribution to variation in seed protein was due to fertilization (*F* = 402.5). In contrast, salinity alone had a relatively minor effect, and the varieties responded similarly to salinity as evidenced by a non-significant interaction. Significant interactions, however, were found in variety × fertilization, as well as in salinity × fertilization, both of which are addressed in later paragraphs. It is worth noting that the two experiments produced different seed protein contents (*F* = 48.09, *P* < 0.001) and an experiment × variety interaction was observed (*F* = 14.94, *P* < 0.001) (data not shown). Upon closer examination, this interaction was caused by variety QQ065, which produced an overall mean protein content of 12.9% in Experiment 1 and 14.9% in Experiment 2. Protein contents of the other three varieties were essentially consistent across the two experiments.

**Table 1:** Analysis of variance with *F*-values for protein content, hardness, and density of quinoa seed.

Effect	*F*-values		
	Protein	Hardness	Density
Model	5.2***	3.6***	2.45***
Variety	24.6***	210.6***	22.82***
Salinity	9.8***	2.0†	2.82*
Fertilization	402.5***	1.1	2.60
Variety × Salinity	1.0	1.0	0.36
Variety × Fertilization	20.6***	10.9***	4.60**
Salinity × Fertilization	3.4**	1.4	0.71
Variety × Salinity × Fertilization	0.8	1.6†	1.55

*†Significant at the 0.10 probability level. ^∗^Significant at the <0.05 probability level. ^∗∗^Significant at the 0.01 probability level. ^∗∗∗^Significant at the <0.001 probability level.*

Across all salinity and fertilization treatments, the variety protein means ranged from 13.0 to 16.7% (data not shown). As expected, high fertilization resulted in an increase in protein content across all varieties. The mean protein contents under high and low fertilization were 15.8 and 13.6%, respectively (**Table 2**). The means of Baer and CO407D were the highest, 15.1 and 14.9%, respectively. QQ065 contained 14.1% protein, significantly lower than the other varieties.

**Table 2:** Salinity level and composition, variety, and fertilization effects on quinoa seed protein content (%).

Salinity	Protein content (%)	Variety	Protein content (%)	Fertilization	Protein content (%)
8 dS m^-1^ NaCl	14.7bc^1^	CO407D	14.9ab	High	15.8a
16 dS m^-1^ NaCl	14.8ab	UDEC-1	14.7b	Low	13.6b
32 dS m^-1^ NaCl	14.9ab	Baer	15.1a		
8 dS m^-1^ Na_2_SO_4_	14.4cd	QQ065	14.1c		
16 dS m^-1^ Na_2_SO_4_	14.2d				
32 dS m^-1^ Na_2_SO_4_	15.2a				

^1^*Different letters in a given column indicate significant differences (P < 0.05).*

Even though salinity effects were relatively smaller than fertilization and variety effects, salinity still had a significant effect on protein content (**Table 1**). The two types of salt exhibited different impacts on protein (**Table 2**). Protein content did not differ according to different concentrations of NaCl with means (across varieties and fertilization levels) from 14.7 to 14.9%. Seed from 32 dS m^-1^ Na_2_SO_4_, however, contained higher protein (15.2%) than that from 8 dS m^-1^ and 16 dS m^-1^ Na_2_SO_4_ (14.4 and 14.2%, respectively).

**FIGURE 1:**
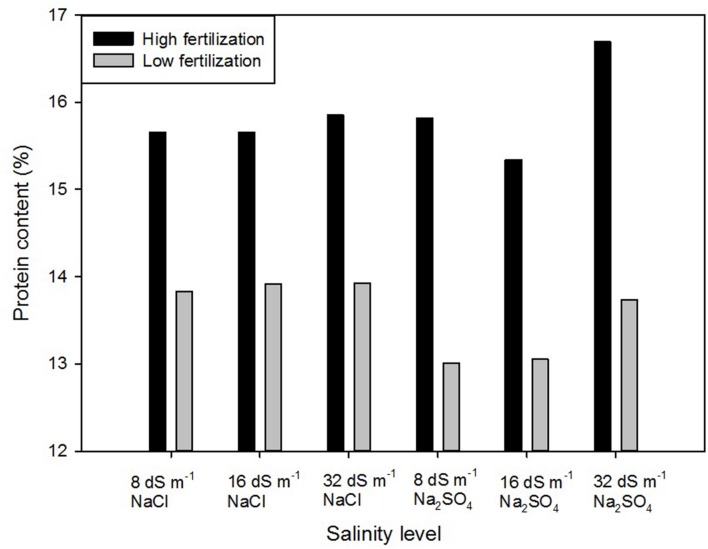
**Protein content (%) of quinoa in response to combined fertility and salinity treatments**.

A significant interaction of salinity × fertilization was detected, indicating that salinity differentially impacted seed protein content under high and low fertilization level (**Figure 1**). Within the high fertilizer treatment, seed protein content from 32dS m^-1^ Na_2_SO_4_ was significantly higher (16.7%) than all other samples, which did not differ from each other (~13%). Within the low fertilizer treatment, seed protein content from 8 dS m^-1^ and 16 dS m^-1^ Na_2_SO_4_ were significantly lower than those from the NaCl treatments and 32dS m^-1^ Na_2_SO_4_.

The significant interaction between variety and fertilization (**Table 1**) was due to the different response of QQ065. Protein mean of QQ065 from high fertilization was 14.4%, lower than the other varieties. CO407D, UDEC-1, and Baer exhibited a decline of 16–18% in protein under low fertilization, while QQ065 dropped only 5%.

### Hardness

Variety exhibited the greatest influence on seed hardness (*F* = 210.6, *P* < 0.001), whereas fertilization did not show any significant effect (**Table 1**). Salinity exhibited a moderate effect (*F* = 2.0, *P* = 0.09). Varieties responded consistently to salinity under various fertilization levels, since neither variety × salinity nor salinity × fertilization interaction was significant. However, a variety × fertilization interaction was observed, which will be discussed in a later paragraph. Similar to the situation in protein content, experiment repetition exhibited a significant influence on seed hardness. Whereas the hardness of CO407D was consistent across the two greenhouse experiments, the hardness of other three varieties all decreased by 8–9%.

Mean hardness was significantly different among varieties. CO407D had the hardest seeds with hardness mean of 10.0 kg (**Table 3**). UDEC-1 was softer at 9.4 kg, whereas Baer and QQ065 were the softest and with similar hardness means of 7.7 and 7.4 kg, respectively.

**Table 3:** Salinity level and composition, variety, and fertilization effects on quinoa seed hardness (kg).

Salinity	Hardness (kg)^1^	Variety	Hardness (kg)
8 dS m^-1^ NaCl	8.3	CO407D	10.0a^2^
16 dS m^-1^ NaCl	8.7	UDEC-1	9.4b
32 dS m^-1^ NaCl	8.5	Baer	7.7c
8 dS m^-1^ Na_2_SO_4_	8.7	QQ065	7.4c
16 dS m^-1^ Na_2_SO_4_	8.9		
32 dS m^-1^ Na_2_SO_4_	8.8		

^1^*Hardness was significant at the 0.09 probability level. ^2^Different letters in a given column indicate significant differences (P < 0.05).*

Salinity exhibited a moderate impact on seed hardness (*P* = 0.09). The highest hardness mean was observed under 16 dS m^-1^ Na_2_SO_4_, whereas the lowest was under 8 dS m^-1^ NaCl, with means of 8.9 and 8.3 kg, respectively.

A significant fertilization × variety interaction was found for seed hardness. The hardness of UDEC-1 and Baer did not differ across fertilization level, whereas CO407D was harder under low fertilization, and QQ065 was harder under high fertilization.

### Seed Density

Variety and salinity both significantly affected seed density, whereas fertilization did not show a significant influence (**Table 1**). The greatest contribution to variation in seed density was due to variety (*F* = 22.82). Salinity exhibited a relatively smaller effect, yet still significant (*F* = 2.82, *P* < 0.05). Neither variety × salinity interaction nor salinity × fertilization interaction was observed, which indicated that varieties similarly responded to salinity under high and low fertilization levels. An interaction of variety × fertilization was found and the details are presented below.

Across all salinity and fertilization treatments, CO407D had the highest mean density, 0.80 g/cm^3^, followed by Baer with 0.69 g/cm^3^ (**Table 4**). UDEC-1 and QQ065 had the lowest and similar densities (~0.65 g/cm^3^).

**Table 4:** Salinity level and composition, variety, and fertilization effects on quinoa seed density (g/cm^3^).

Salinity	Density (g/cm^3^)	Variety	Density (g/cm^3^)
8 dS m^-1^ NaCl	0.69bcˆ1	CO407D	0.80a
16 dS m^-1^ NaCl	0.68bc	UDEC-1	0.66bc
32 dS m^-1^ NaCl	0.71abc	Baer	0.69b
8 dS m^-1^ Na_2_SO_4_	0.66c	QQ065	0.65c
16 dS m^-1^ Na_2_SO_4_	0.74a		
32 dS m^-1^ Na_2_SO_4_	0.72ab		

^1^*Different letters in a given column indicate significant differences (P < 0.05).*

With regard to salinity effect, the Na_2_SO_4_ treatments exhibited differential influence on seed density. Density means did not significantly change due to the increased concentration of NaCl, which ranged from 0.68 to 0.71 g/cm^3^ (**Table 4**). The samples from 8 dS m^-1^ Na_2_SO_4_ soil had lower density (0.66 g/cm^3^) than those from 16 dS m^-1^ and 32 dS m^-1^ Na_2_SO_4_, 0.74 and 0.72g/cm^3^, respectively.

A significant variety × fertilization interaction was found. With closer examination, UDEC-1 and Baer yielded higher density seeds under high fertilization, whereas CO407D and QQ065 did not differ in density between fertilization treatments.

### Correlations of Protein, Hardness, and Density

Correlation coefficients among seed protein content, hardness, and density are shown in **Table 5**. No significant correlation was detected between protein content and seed hardness. However, both protein content and hardness were correlated with seed density. The overall correlation coefficient was low (*r* = 0.19, *P* = 0.03) between density and protein. A marginally significant correlation was found between density and protein content of the seeds from NaCl salinized soil under low fertilization. No correlation was found between density and protein content of the seeds from NaCl salinized soil under high fertilization, or Na_2_SO_4_ salinized soil.

**Table 5:** Correlation coefficients of protein, hardness, and density of quinoa seed.

Correlation	All	NaCl		Na_2_SO_4_	
		High fertilization	Low fertilization	High fertilization	Low fertilization
Protein – Density	0.19^∗^	0.13^ns^	0.29†	0.26^ns^	0.19^ns^
Hardness – Density	0.38^∗∗∗^	0.27^ns^	0.51^∗∗∗^	0.22^ns^	0.47^∗∗∗^

*ns, Not significant. †Significant at the 0.10 probability level. ^∗^Significant at the <0.05 probability level. ^∗∗∗^Significant at the <0.001 probability level.*

The overall correlation coefficient was *r* = 0.38 (*P <* 0.0001) between density and hardness. The low fertilization samples from both NaCl and Na_2_SO_4_ soil showed significant correlations between density and hardness, with coefficients of *r* = 0.51 and 0.47 (both *P <* 0.005). The high fertility samples did not exhibit any correlation between density and hardness.

### Correlation with Yield, Leaf Greenness Index, Plant Height, and Seed Minerals Contents

Correlation between seed quality and yield, leaf greenness index, plant height, and seed mineral concentration were obtained using data from Peterson and Murphy (2015) (**Table 6**). Seed hardness was significantly correlated with yield and plant height (*r* = 0.35 and 0.31, respectively). Protein content and density, however, did not correlate with yield, leaf greenness, or plant height. Correlations were found between quality indices and the concentration of different minerals. Protein was negatively correlated with Cu and Mg (*r* = -0.52 and -0.50, respectively). Hardness was negatively correlated with Cu, P, and Zn (*r* = -0.37, -0.56, -0.29, respectively), but was positively correlated with Mn (*r* = 0.57). Density was negatively correlated with Cu (*r* = -0.35).

**Table 6:** Correlation coefficients of quinoa seed quality, and agronomic performance and seed mineral content^a^.

	Protein	Hardness	Density
Yield	0.04	0.35*	0.06
Plant height	-0.04	0.31*	0.11
Cu	-0.52***	-0.37**	-0.35*
Mg	-0.50***	0.04	0
Mn	-0.06	0.57***	0.25†
P	-0.01	-0.56***	-0.15
Zn	-0.04	-0.29*	-0.28†

^a^*Agronomic and mineral contents taken from Peterson and Murphy (2015). †Significant at the 0.10 probability level. ^∗^Significant at the <0.05 probability level. ^∗∗^Significant at the 0.01 probability level. ^∗∗∗^Significant at the <0.001 probability level.*

## Discussion

### Protein

Although salinity exhibited a significant effect on seed protein content, the impact was relatively minor compared to fertilization and variety effects. In another words, over a wide range of saline soil, quinoa can grow and yield seeds with relatively stable protein content.

Protein content of quinoa growing under salinized soil ranged from 12.7 to 16.7% (data not shown), within the general range of protein content under non-saline conditions, which was 12–17% (Rojas et al., 2015). Saline soil did not cause a significant decrease in seed protein. It is interesting to notice that the samples from 32 dS m^-1^ Na_2_SO_4_ tended to contain the highest protein, especially in variety QQ065. The studies of Karyotis et al. (2003) and Koyro and Eisa (2008) also indicated that protein content significantly increased under high salinity (NaCl) whereas total carbohydrate decreased. In contrast, Ruffino et al. (2010) found that quinoa protein decreased under 250 mM NaCl salinity in a growth chamber experiment. It is reasonable to conclude that salinity exhibits contrasting effects on different quinoa genotypes.

Na_2_SO_4_ level exhibited a significant influence on protein content, whereas NaCl level did not. In the study of Koyro and Eisa (2008), however, seed protein content of the quinoa variety Hualhuas (origin Peru) increased under the highest salinity level of 500 mM NaCl compared to lower NaCl levels (0 – 400 mM). This disagreement of NaCl influence may be due to diversity of genotypes. It is worth noting that quinoa protein contents in this paper were primarily above 13% based on a wet weight basis (as-is-moisture of approximately ~8–10%) even under saline soil and low fertilization level. This protein content is generally equal to or higher than that of other crops such as barley and rice (Wu, 2015). In conclusion, quinoa maintained high and stable protein content under salinity stress.

Besides seed quality, grain yield is another significant factor when quinoa is grown in marginal environments. The yield data were reported in the previous study (Peterson and Murphy, 2015). Under 32 dS m^-1^ Na_2_SO_4_, the yield of CO407D, UDEC-1, and Baer decreased by 24.5, 10.8, and 11.6%, respectively, and the yield decline was much lower than a barley variety Albacete (yield decline of 82.4%). The yield decline of QQ065 was 54.5% under 32 dS m^-1^ Na_2_SO_4_. In the same soil condition, both CO407D and QQ065 exhibited increased protein content. Hence, if only protein content is considered, QQ065 contained the highest protein under high Na_2_SO_4_; however, if both yield and protein content are considered, then CO407D is the variety more suitable for severe Na_2_SO_4_ affected areas since it exhibited a moderate yield decline and a significant increase in protein content. It also implies the importance to evaluate both agronomy performance and seed quality when studying crops’ adaption to extreme environments.

### Hardness

Quinoa seed hardness was only moderately affected by salinity (*P* = 0.09), indicating that quinoa primarily maintained seed texture when growing under a wide range of saline soil. CO407D exhibited the hardest seed (10.0 kg), whereas Baer and QQ065 were relatively soft (7.4 – 7.7 kg). A previous study indicated a hardness range of 5.8 – 10.9 kg among 11 quinoa varieties and 2 commercial samples (Wu et al., 2014). The commercial samples had hardness values of 6.2 and 7.1 kg. Since commercial samples generally maintain stable quality and indicate an acceptable level for consumers, seed hardness around 7 kg, as in Baer and QQ065, should be considered as acceptable quality. The hardness of CO407D was close to that of the colored variety ‘Black’ (10.0 kg), which had a thicker seed coat than that of the yellow seeded varieties. It was reported that a thicker seed coat is related to harder texture (Fraczek et al., 2005).

Even though the greenhouse is a highly controlled environment, and the two experiments were conducted in similar seasons (planted in September and October, respectively), seed protein and hardness were nevertheless different across the two experiments. However, ANOVA indicated modest-to-no significant interactions with salinity and fertilization such that responses to salinity and fertilization were consistent with little or no change in rank order. Even though the experiment × variety interaction was significant, the *F*-values were relatively low compared to the major effects such as variety and fertilization, and neither of them exhibited cross-over interaction. This is a particularly noteworthy result for breeders, farmers, and processors.

### Density

The range of seed density under salinity, 0.55 – 0.89 g/cm^3^, was comparable to the density range of 13 quinoa samples (0.58 – 0.76 g/cm^3^) (Wu et al., 2014). Generally, CO407D had higher seed density (0.71 – 0.89 g/cm^3^), which indicated that seed density of this variety was affected by salinity stress. In contrast, the density of QQ065 did not change according to salinity type or concentration, which indicated a stable quality under saline soil.

### Correlations

The correlation between seed hardness and density was only significant under low fertilization, but not under high fertilization. The high fertilization level in the greenhouse experiment exceeded the amount of fertilizer that would normally be applied in field environments, whereas the low fertilization level was closer to the field situation. Therefore, correlation between hardness and density may still exist in field trials.

## Conclusion

This study confirmed quinoa’s tolerance to salinity stress in terms of seed quality. Under saline soil conditions, quinoa did not show any marked decrease in seed quality such as protein content, hardness, and density. Protein content even increased under high Na_2_SO_4_ concentration (32 dS m^-1^). Varieties exhibited great differential reactions to fertilization and salinity levels. QQ065 maintained a similar level of hardness and density, whereas seed of CO407D was both harder and higher density under salinity stress. If only seed quality is considered, then QQ065 is the most well-adapted variety in this study. Additionally, the influences of NaCl and Na_2_SO_4_ were different. The higher concentration of Na_2_SO_4_ tended to increase protein content and seed density, whereas NaCl concentration did not exhibit any significant difference on those quality indexes. In other words, quinoa can be grown in areas severely affected by Na_2_SO_4_ and still produce high protein seeds, which can be a good protein resource for vegetarians or malnourished populations.

In the present study, protein content, seed hardness, and density were selected as quinoa seed quality indexes due to their significance in nutritional value and end-use quality. Besides those factors, other seed components such as essential amino acids, starch, fiber, and minerals are also important to nutrition value. Their variation under different salt types should be investigated in the future. Hence, when quinoa is applied to a specific salinity area, the right varieties can be utilized not only with the consideration of yield but also the health benefits to local people. When addressing malnutrition in developed countries, the profile of essential amino acids, protein quality (such as protein efficiency ratio), and deficient minerals should be specifically evaluated. Furthermore, this study was based on greenhouse experiments. The soil conditions and environment in the field are much more complex than the soil medium used in the greenhouse. Based on the conclusion from this research, further field study should be conducted using the varieties best adapted to specific salt type and salinity level.

## Author Contributions

AP set up the experiment design in the greenhouse and grew, harvested, and processed quinoa samples. GW collected seed quality data such as protein content, seed hardness, and density. AP and GW together processed the data. GW also drafted the manuscript. KM and CM edited the manuscript.

## Conflict of Interest Statement

The authors declare that the research was conducted in the absence of any commercial or financial relationships that could be construed as a potential conflict of interest.
